# Processing Discontinuous Chinese Separable Words: Evidence from Eye Tracking

**DOI:** 10.3390/bs16081285

**Published:** 2026-07-28

**Authors:** Ao Sun, Yulin Yuan

**Affiliations:** 1 School of Chinese as a Second Language, Peking University, Beijing 100871, China; 2 Faculty of Arts and Humanities, University of Macau, Macau 999078, China

**Keywords:** separable words, discontinuous forms, eye tracking, verb–object phrases, quasi-separable words, contextual surprisal

## Abstract

Chinese separable words combine lexical integrity with structural separability, but how readers process discontinuous forms during sentence reading remains unclear. Thirty native Chinese speakers read sentences containing discontinuous separable words, verb–object (V–O) phrases, or quasi-separable words (QSWs) while their eye movements were recorded. Corpus-derived discontinuous-structure frequency and local contextual surprisal were included as continuous predictors. Separable words did not differ reliably from V–O phrases in acceptability judgments or target-region eye-movement measures, suggesting no stable additional processing cost relative to the V–O baseline. QSWs were accepted less often, elicited longer judgment response times, and showed greater target-region processing difficulty. Within the QSW condition, higher frequency was associated with shorter response times and greater acceptance. After frequency and surprisal were added, all adjusted pairwise comparisons for the binary fixation measures were nonsignificant. For conditional durations, reliable adjusted differences were limited to V–O phrases versus QSWs; separable words did not differ reliably from either comparison condition. Higher surprisal predicted greater second-fixation probability. Together, these findings suggest that discontinuous separable words benefit from distributional and constructional support, allowing their components to enter V–O-like integration without obligatory lexical reconstruction.

## 1. Introduction

### 1.1. Chinese Separable Words

Across languages, some fixed expressions permit both adjacent and discontinuous realizations. English verb–particle constructions allow either order with little change in meaning, as shown in Example (1) (Lohse et al., 2004; Wisintainer & Mota, 2018). German separable verbs similarly separate the verbal stem and particle in main clauses but combine them in subordinate clauses, as shown in Example (2) (Czypionka et al., 2019; Müller, 2002; Smolka et al., 2019). Related patterns have also been described in Dutch (Behrens, 1998; Booij, 1990).

(1) a. Eat up the candy.
  b. Eat the candy up.
(2) a. Main clause
ErfährtdenBaumum.herunsthetreeoverHe runs over the tree.
  b. Subordinate clause
wennerdenBaumumfährtifhethetreeover.runsif he runs over the tree.

Such expressions raise the question of whether they are stored as holistic lexical units or assembled online from their components (Cappelle et al., 2010; Tiv et al., 2019). Chinese separable words, or *liheci*, are particularly informative because they commonly occur as word-level units in their combined forms, yet their internal components may include bound morphemes that cannot ordinarily function as independent words. Example (3) illustrates this property.

(3) a.
他今天下午洗澡*Ta**jintian**xiawu**xi-zao*HetodayafternoonbathHe will take a bath this afternoon.
  b.
他今天下午洗了一次澡*Ta**jintian**xiawu**xi**le**yi-ci**zao*HetodayafternoonwashPERFone-CLbathHe took a bath this afternoon.
  c.
*他今天下午一次洗澡了*Ta*jintian**xiawu**yi-ci**xi-zao**le*Hetodayafternoonone-CLbathPERFNote. The asterisk indicates that the sentence is unacceptable. Glossing abbreviations: PERF = perfective aspect; CL = classifier.

In Example (3a), *xi-zao* ‘take a bath’ occurs as a word in its combined form. Its second component, *zao* ‘bath’, does not ordinarily function as an independent word and occurs primarily within compounds. When the sentence expresses event-related information such as completion or frequency, however, the aspect marker *le* and the quantificational expression *yi-ci* ‘once’ must intervene between the two components, yielding the discontinuous form *xi-le yi-ci zao* in (3b). These elements cannot simply be placed before or after the combined word, as shown by the unacceptable sentence in (3c). Chinese separable words thus have relatively stable lexical meanings while permitting internal expansion in appropriate syntactic contexts.

This combination of lexical boundness and syntactic separability distinguishes Chinese separable words from the English verb–particle constructions and German separable verbs discussed above. In English and German, the separated components generally have greater syntactic independence. In Chinese separable words, by contrast, components that are not normally available as free words may nevertheless occur separately and participate in syntactic organization. Separable words therefore instantiate a particularly complex relationship between lexicalization and syntactic combinability, making them a useful test case for theories of lexical representation, component accessibility, and online structural integration.

The structural status of Chinese separable words has long been debated. One line of analysis emphasizes their lexical properties. From this perspective, their semantic integrity, bound internal components, and restricted expansion patterns support treating them as lexical units despite their surface discontinuity. Discontinuous forms are consequently analyzed as surface realizations derived through syntactic or morphological operations such as copying, movement, or complementary deletion (Chao, 1968; Guo, 2017; Pan & Ye, 2015; Ye & Pan, 2018; Zhao & Zhang, 1996).

A second line of analysis emphasizes their constructional properties and syntactic behavior. On this account, separable words are closely related to the verb–object construction, and their discontinuous forms resemble ordinary verb–object phrases in their expansion patterns. They need not, therefore, be forced into a strict word-versus-phrase dichotomy (Lü, 1979; Packard, 2000; Zhu, 1982). Constructional approaches have analyzed discontinuous separable words as products of constructional embedding or restructuring within an existing network (Cao, 2016), or as form–meaning mappings realized through formal metonymy in the verb–object construction (Yuan, 2018). Under these analyses, the two components can enter the syntactic structure and combine with intervening material without requiring a separate stage of lexical restoration.

These theoretical debates have motivated psycholinguistic research on the representation of Chinese separable words in the mental lexicon and their online processing. Central questions include whether separable words are accessed primarily as holistic lexical units, whether their internal components become available during processing, and how their mixed lexical and phrasal properties are reflected in comprehension.

### 1.2. Psycholinguistic Studies on Chinese Separable Words

Psycholinguistic studies of Chinese separable words vary substantially in paradigm, material type, and operational definition, and their findings have not converged on a unified account. Zhang and Jiang (2010) used event-related potentials (ERPs) to compare compounds, separable words, and verb–object phrases in their combined forms. Separable words patterned more closely with phrases than with compounds in the P2 and N400 components, but their P600 response differed from both comparison conditions. Zhang and Jiang therefore argued that combined separable words exhibit a processing profile intermediate between those of words and phrases.

Gu et al. (2011) further examined the role of component boundness. Separable words composed of two free morphemes and those composed of a free morpheme plus a bound morpheme produced different patterns in ERP components associated with semantic and syntactic processing. Using a similar ERP paradigm, however, Gu et al. (2018) found no significant N400 or P600 differences and observed distinctions only at earlier stages, including the P200. Together, these results suggest that component properties and material selection may affect separable-word processing, but the available ERP evidence remains mixed.

Behavioral studies have likewise examined separable words and related structures. In a semantic-transparency judgment task, Han et al. (2024) found no significant difference between separable and nonseparable words, although both differed from phrases. Xia and Wang (2024) used a lexical-decision task to investigate spacing effects in verb–complement separable words. Processing varied with structural tightness, but the overall pattern favored a continuum rather than a categorical distinction. More recently, X. Chen et al. (2025) used repeated-priming lexical decision to examine Chinese two-character verb–object constructions. Across two experiments, separability interacted with whether the object component was free or bound. Their results indicate that skilled readers do not rely exclusively on whole-word representations but are also sensitive to the syntactic and semantic relationships between components.

Taken together, the existing evidence suggests that separable-word processing is shaped by component properties, structural tightness, and separability rather than by whole-word access alone. Most previous studies, however, have examined combined forms in relatively constrained tasks, leaving the processing of discontinuous forms during sentence reading largely unexplored. Distributional predictors such as construction frequency and contextual predictability have also seldom been incorporated into the statistical analysis. The present study therefore asked whether processing differences among discontinuous separable words, verb–object phrases, and quasi-separable words are associated with distributional statistics.

### 1.3. The Current Study

To address this gap, we conducted an eye-tracking experiment comparing discontinuous separable words, ordinary verb–object (V–O) phrases, and quasi-separable words (QSWs). V–O phrases served as a freely compositional baseline, whereas QSWs were included as a low-conventionality comparison condition because they are lexicalized disyllabic units whose discontinuous uses are weakly established and were expected to be less acceptable than those of typical separable words.

The study addressed two questions. First, do discontinuous separable words show processing costs beyond those observed for V–O phrases? Dependency-integration and cue-based retrieval accounts identify integration distance and retrieval demands as potential sources of comprehension difficulty (Gibson, 1998; Lewis & Vasishth, 2005; Phillips & Lewis, 2013). A reliable residual difference between separable words and V–O phrases would therefore be compatible with distinct integration demands, whereas a similar processing profile would be consistent with shared V–O-like integration. Second, to what extent is discontinuous processing associated with distributional experience? Usage-based and expectation-based accounts predict easier processing for structures that are more frequent and more predictable in context (J. L. Bybee, 2013; Hale, 2001; Kuperberg & Jaeger, 2016; Levy, 2008; Pickering & Gambi, 2018).

Accordingly, if discontinuous separable words are processed primarily through general V–O integration supported by distributional information, they should pattern closely with ordinary V–O phrases. Any differences that remain after the distributional predictors are included would be consistent with additional structural constraints, although the observational nature of these predictors precludes a causal interpretation. Because discontinuous QSWs are infrequent, weakly predictable, and low in conventionality, they were expected to be more difficult to process; we further tested whether this difficulty covaried with discontinuous-structure frequency and contextual surprisal.

## 2. Materials and Methods

### 2.1. Participants

Thirty native speakers of Chinese participated in the experiment, including 13 men. Their mean age was 21 years (*SD* = 1.2, range = 19–24). All were students whose majors were neither linguistics nor psychology. They were right-handed, had normal or corrected-to-normal vision, and reported no history of neurological or psychiatric disorders. None of the eye-tracking participants had taken part in the familiarity or acceptability rating tasks used to select the materials. All participants provided written informed consent before the experiment and received compensation upon completion. The Ethics Committee of the School of Chinese as a Second Language, Peking University, reviewed the study documentation and determined that formal ethical review was not required.

### 2.2. Materials

The experiment compared three types of discontinuous expressions: separable words, V–O phrases, and QSWs. Previous studies have noted that discontinuous use in Mandarin shows an expanding tendency, with some lexical items outside the prototypical class of separable words also occurring in separated forms (Wang, 2011; Fang, 2020). In the present study, QSWs were defined operationally rather than as a sharply bounded lexical class. They were selected as attested but non-prototypical cases of discontinuous use: each QSW item had attested discontinuous uses in the BCC Corpus (Xun et al., 2016) or in the online corpus survey reported by Zhuang (2020), and was therefore distinguished from newly created pseudo-forms. At the same time, these items were not treated as typical separable words because most of them were not marked as separable words in the *Modern Chinese Dictionary* (Dictionary Editing Office, Institute of Linguistics, Chinese Academy of Social Sciences, 2016) and because their discontinuous uses were judged substantially less acceptable in the norming task. Thus, the QSW condition was defined by a combination of attestation, lexicographic status, acceptability, and degree of conventionalization, rather than by corpus frequency alone. This condition was included as a low-conventionality comparison condition, allowing us to compare typical, highly conventionalized separable-word uses in discontinuous form with non-prototypical but attested discontinuous uses.

We constructed 40 matched candidate sets, yielding 120 sentences, with one sentence per condition in each set. Each target expression contained a single monosyllabic aspect marker or classifier between its two components and occurred at the end of the first clause in a two-clause sentence. The conditions were matched for sentence length, target position, and the length of the intervening element.

Before the eye-tracking experiment, two rating tasks assessed lexical familiarity and sentence acceptability. In the familiarity task, 20 native Chinese speakers who did not participate in the eye-tracking experiment rated the expressions from the 40 candidate sets on a 7-point scale, where 1 indicated ‘very unfamiliar’ and 7 indicated ‘very familiar’. In the acceptability task, a different group of 20 native Chinese speakers rated the 120 candidate sentences on a 10-point scale, where 1 indicated ‘completely unacceptable’ and 10 indicated ‘completely acceptable’.

On the basis of these ratings, 30 matched sets were selected for the formal experiment, yielding 90 critical sentences (30 per condition). For each rating task, responses were first averaged across the 20 raters for each item; the resulting item-level means were then analyzed across the 30 matched sets using a one-way repeated-measures analysis of variance (ANOVA), with condition as the within-set factor. Mean familiarity ratings were 6.29 (SD = 0.50) for separable words, 6.44 (SD = 0.34) for V–O phrases, and 6.26 (SD = 0.40) for QSWs. The effect of condition was not significant, F(2, 58) = 2.34, *p* = 0.105, η^2^p = 0.075. Mean sentence-acceptability ratings were 8.91 (SD = 0.41), 8.72 (SD = 0.33), and 3.98 (SD = 1.91), respectively. The condition effect was significant. Because sphericity was violated, Mauchly’s W = 0.173, χ^2^(2) = 49.07, *p* < 0.001, Greenhouse–Geisser-corrected results are reported, F(1.09, 31.75) = 183.69, *p* < 0.001, η^2^p = 0.864. Bonferroni-adjusted comparisons showed no significant difference between the separable-word sentences and the V–O phrase sentences, *p* = 0.105, whereas QSW sentences received significantly lower ratings than both other conditions, *p*s < 0.001. Thus, the separable-word and V–O phrase conditions were closely matched in familiarity and acceptability, whereas the QSW condition represented a low-acceptability discontinuous pattern. Corpus-derived frequency and surprisal were subsequently included to assess whether processing difficulty covaried with distributional support.

In addition to the 90 critical sentences, the experiment included 30 anomalous sentences and 60 filler sentences. Table 1 presents representative materials.

### 2.3. Apparatus and Procedure

Eye movements were recorded at 1000 Hz using an EyeLink 1000 Plus eye tracker (SR Research, Mississauga, ON, Canada). Participants were seated approximately 60 cm from the display, with their heads stabilized by a chin-and-forehead rest. A practice session preceded the formal experiment.

The experiment comprised three blocks of 60 trials. Each block contained 30 critical sentences (10 per condition), 10 anomalous sentences, and 20 fillers. Trials were randomized within blocks, and the three variants from each matched set were assigned to different blocks. A nine-point calibration was performed at the beginning of each block.

Each trial began with a fixation marker at the sentence-initial position. After 800 ms, the sentence appeared in 32-point white SimSun font against a dark-gray background. Participants read at their own pace and pressed the space bar when they had finished. They then judged whether the sentence was acceptable. Judgment response time was measured from the onset of the response screen to the keypress. Responses were coded as acceptable or unacceptable; no predetermined correct response was assigned to QSW sentences. Response-key mapping was counterbalanced across participants. The experiment lasted approximately 15 min.

### 2.4. Data Preprocessing and Statistical Analysis

Eye-movement data were preprocessed using DataViewer (version 5.1.1). Analyses focused on the target region containing the second component of each discontinuous expression, because the main question concerned its integration with the preceding verbal component. For example, in *Li Ming zai shanli chi-guo ku* ‘Li Ming had endured hardship in the mountains’, *ku* ‘hardship’ constituted the target region.

For the behavioral analyses, trials with missing, nonfinite, nonpositive, or judgment response times shorter than 100 ms were excluded, leaving 2654 trials. Judgment response times were log-transformed and analyzed using linear mixed-effects models (LMMs), whereas acceptable versus unacceptable judgments were analyzed using binomial-logit generalized linear mixed-effects models (GLMMs).

For the eye-movement analyses, trials with substantial track loss that prevented reliable assignment of fixations to the target region were excluded. After this trial-level exclusion, 2531 of the 2700 critical trials remained. These trials were analyzed irrespective of the participant’s acceptability judgment. Because the target region was typically a single Chinese character and was frequently skipped, we analyzed both binary and conditional duration measures. Within the retained trials, target-region fixations shorter than 80 ms or longer than 1000 ms were removed (Jegerski & Fernández Cuenca, 2025; Slattery et al., 2011; Yan et al., 2013). Binomial-logit GLMMs examined any-fixation probability and second-fixation probability. LMMs examined log-transformed first-fixation duration and dwell time, conditional on the availability of a valid positive duration.

Discontinuous-structure frequency and local contextual surprisal were calculated from the multidomain component of the BCC Corpus and entered as continuous predictors. Discontinuous-structure frequency indexed the overall corpus experience with the separated use of a given V–N combination and was defined as the number of attested tokens in which the first verbal component V and the second component N occurred non-adjacently with any intervening material, schematized as V + M + N. Local contextual surprisal was computed for the specific experimental context, P(N|V + m) = f(V + m + N)/f(V + m), where m denotes the actual intervening element in that sentence. Surprisal was defined as −log[P(N|V + m) + 0.00001], following the general definition of surprisal as the negative logarithm of the probability of an upcoming element given its preceding context (Hale, 2001; Levy, 2008; Smith & Levy, 2013). Concordance lines were manually checked to exclude homographic and contextually irrelevant occurrences. Frequency was transformed as log(frequency + 1), and log frequency and surprisal were standardized across the 90 critical items before being merged with the trial-level datasets.

All analyses were conducted in R version 4.4.0 (R Core Team, 2024). LMMs and binomial-logit GLMMs were fitted using the lme4 package (Bates et al., 2015). Approximate degrees of freedom and *p* values for LMM coefficient summaries were obtained using lmerTest (Kuznetsova et al., 2017), and estimated marginal means, pairwise comparisons, and simple-slope analyses were conducted using emmeans (Lenth & Piaskowski, 2026).

The full fixed-effects structure included condition, standardized log frequency, standardized surprisal, and the interactions between condition and each distributional predictor. Separable words served as the reference condition. Random-effects structures were determined separately for each dependent variable. The judgment-response-time model included random intercepts for participants and items. The acceptability model included random intercepts for participants and items, together with by-participant random slopes for condition. For the eye-movement models, random intercepts for participants and items were included whenever supported by the data. When the estimated item-intercept variance was zero or near zero, resulting in singular fits, the unsupported item random intercept was removed and all models within the relevant nested comparison were refitted with the same simplified random-effects structure.

The omnibus contributions of fixed effects were evaluated using likelihood-ratio tests comparing nested models. LMMs involved in these comparisons were fitted using maximum likelihood rather than restricted maximum likelihood. All models within a comparison were fitted to identical observations and used the same random-effects structure. Pairwise comparisons were Tukey-adjusted. For models containing continuous predictors, pairwise comparisons were evaluated at standardized log frequency = 0 and standardized surprisal = 0.

As an exploratory check of whether condition-related differences emerged before readers reached the target second component, we analyzed two pre-target regions: the Verb region and the intervening-element region. These analyses used the same 2531 cleaned trials as the main target-region analyses. For each region, any-fixation probability and first-fixation duration were analyzed using condition-only mixed-effects models with random intercepts for participants and items.

Additional item-level diagnostics examined condition-predictor associations and the observed ranges of the standardized predictors by condition. We also conducted exploratory sensitivity analyses to examine whether QSW target-region eye movements were associated with participants’ subsequent acceptability judgments. First, within the QSW condition, we compared trials that were subsequently accepted with those that were rejected, controlling for standardized log frequency and surprisal. Second, as a robustness check, we repeated the additive condition analyses after retaining only trials judged acceptable by participants. Because acceptability judgments were made after sentence reading, these analyses were interpreted descriptively rather than as causal tests. Fixed-effect coefficient tables for the additive eye-movement models and full results of the diagnostic, pre-target-region, and sensitivity analyses are provided in the Supplementary Materials.

## 3. Results

### 3.1. Behavioral Results

Descriptive statistics for the behavioral measures and corpus-based predictors are presented in Table 2. Sentences containing separable words and V–O phrases received similarly high acceptance rates, at 96.8% and 98.2%, respectively, and elicited comparable mean judgment response times of 532 and 515 ms. By contrast, QSW sentences were judged acceptable on 49.8% of trials and elicited a substantially longer mean judgment response time of 1461 ms. The conditions also differed markedly in their distributional properties. The mean raw target-structure frequency was 8 in the QSW condition, compared with 794 for separable words and 1539 for V–O phrases. QSW items also had substantially higher surprisal, indicating lower contextual predictability of the target component. Additional item-level diagnostics confirmed that condition was strongly associated with the distributional predictors. One-way ANOVAs showed large condition effects for log frequency, F(2, 87) = 147.28, *p* < 0.001, η^2^ = 0.772, and surprisal, F(2, 87) = 67.72, *p* < 0.001, η^2^ = 0.609. The two distributional predictors were also strongly correlated across all 90 critical items, r = −0.762, *p* < 0.001. Although the grand-mean covariate values used for adjusted pairwise comparisons—z-log frequency = 0 and z-surprisal = 0—fell within the marginal observed range of each condition, overlap was limited, especially for QSW frequency. Full item-level diagnostics are provided in Tables S3 and S4.

#### 3.1.1. Judgment Response Times

Judgment response times were analyzed using an LMM with log-transformed response time as the dependent variable. The fixed-effects structure included condition, standardized log frequency, standardized surprisal, and the interactions between condition and each distributional predictor. The model included random intercepts for participants and items.

Likelihood-ratio comparisons showed that the addition of condition and its interactions with the two distributional predictors significantly improved model fit, χ^2^(6) = 29.48, *p* < 0.001. The addition of standardized log frequency and its interactions with condition also significantly improved model fit, χ^2^(3) = 10.86, *p* = 0.013, whereas standardized surprisal and its interactions did not reach conventional significance, χ^2^(3) = 6.37, *p* = 0.095. The four condition-by-predictor interaction terms were jointly significant, χ^2^(4) = 17.96, *p* = 0.001, indicating that the associations between the distributional predictors and judgment response time varied across conditions.

The interaction between standardized log frequency and the QSW condition was significant, estimate = −0.521, *SE* = 0.159, *t* = −3.28. Simple-slope analyses showed that higher frequency was associated with shorter judgment response times in the QSW condition, slope = −0.423, *t* = −3.21, *p* = 0.002. The corresponding frequency slopes were not significant in the separable-word condition, slope = 0.098, *t* = 1.11, *p* = 0.272, or in the V–O phrase condition, slope = −0.008, *t* = −0.07, *p* = 0.941.

#### 3.1.2. Acceptability Judgments

The probability of an acceptable judgment was analyzed using a binomial-logit GLMM. The fixed-effects structure was the same as that of the response-time model. The model included random intercepts for participants and items, together with by-participant random slopes for condition.

Likelihood-ratio comparisons showed that the addition of condition and its interactions with the two distributional predictors significantly improved model fit, χ^2^(6) = 23.16, *p* < 0.001. Standardized log frequency and its interactions with condition also made a significant contribution, χ^2^(3) = 7.95, *p* = 0.047, whereas standardized surprisal and its interactions did not, χ^2^(3) = 1.45, *p* = 0.694. The four condition-by-predictor interaction terms were not jointly significant, χ^2^(4) = 8.49, *p* = 0.075.

Simple-slope analyses showed that higher frequency was associated with a greater probability of an acceptable judgment in the QSW condition, slope = 2.038, *z* = 2.63, *p* = 0.009. The corresponding frequency slopes were not significant in the separable-word condition, slope = 0.407, *z* = 0.64, *p* = 0.522, or in the V–O phrase condition, slope = −0.711, *z* = −0.79, *p* = 0.433. Although the frequency slope was significant in the QSW condition, the interaction terms were not jointly significant; therefore, the evidence that the frequency association differed reliably across conditions was limited. Fixed-effect estimates for both behavioral models are presented in Table 3.

### 3.2. Eye-Tracking Results

Descriptive statistics for the target-region eye-movement measures are presented in Table 4. Separable words and V–O phrases showed similar descriptive patterns, with any-fixation probabilities of about one-third and relatively low second-fixation probabilities. QSWs showed higher any-fixation and second-fixation probabilities, at 52.3% and 17.2%, respectively, as well as longer first-fixation durations and dwell times among trials with valid duration measures.

#### 3.2.1. Binary Fixation Measures

The binary fixation analyses included all 2531 retained eye-movement trials. Condition was significant in the condition-only models for both any-fixation probability, χ^2^(2) = 34.84, *p* < 0.001, and second-fixation probability, χ^2^(2) = 83.25, *p* < 0.001. After standardized log frequency and surprisal were added, the condition effect was no longer significant for either any-fixation probability, χ^2^(2) = 0.48, *p* = 0.787, or second-fixation probability, χ^2^(2) = 2.11, *p* = 0.348.

For any-fixation probability, higher frequency was associated with a lower probability of fixation, χ^2^(1) = 4.55, *p* = 0.033, whereas surprisal was not significant, χ^2^(1) = 2.30, *p* = 0.130. For second-fixation probability, the frequency effect did not reach conventional significance, χ^2^(1) = 3.35, *p* = 0.067, whereas higher surprisal was associated with a greater probability of a second fixation, χ^2^(1) = 7.19, *p* = 0.007. The condition-by-predictor interactions were not significant for either any-fixation probability, χ^2^(4) = 4.86, *p* = 0.302, or second-fixation probability, χ^2^(4) = 1.06, *p* = 0.901.

Pairwise comparisons from the full interaction models are presented in Table 5. At the mean levels of the standardized predictors, none of the pairwise condition comparisons was significant for either any-fixation probability or second-fixation probability.

#### 3.2.2. Conditional Fixation Durations

The conditional duration analyses included 1001 observations for first-fixation duration and 1011 for dwell time. Condition was significant in the condition-only models for both first-fixation duration, χ^2^(2) = 21.12, *p* < 0.001, and dwell time, χ^2^(2) = 48.39, *p* < 0.001. After standardized log frequency and surprisal were added, the condition effect remained significant for first-fixation duration, χ^2^(2) = 6.55, *p* = 0.038, but was not significant for dwell time, χ^2^(2) = 4.90, *p* = 0.086.

Neither frequency nor surprisal made a reliable independent contribution to first-fixation duration: frequency: χ^2^(1) = 0.48, *p* = 0.490; surprisal: χ^2^(1) = 0.01, *p* = 0.928. The corresponding effects were also not significant for dwell time: frequency: χ^2^(1) = 0.00, *p* = 0.955; surprisal: χ^2^(1) = 3.49, *p* = 0.062. The condition-by-predictor interactions were not significant for either first-fixation duration, χ^2^(4) = 7.09, *p* = 0.131, or dwell time, χ^2^(4) = 4.23, *p* = 0.376.

Pairwise comparisons from the full interaction models are presented in Table 6. At the mean levels of the standardized predictors, V–O phrases had shorter durations than QSWs for both first-fixation duration, difference = −0.250 on the log scale, *p* = 0.003, and dwell time, difference = −0.263 on the log scale, *p* = 0.043. Separable words did not differ reliably from either V–O phrases or QSWs on either duration measure.

Overall, separable words and V–O phrases did not differ reliably on any target-region measure. QSWs showed greater processing difficulty descriptively, but after frequency and surprisal were added, the omnibus condition effects and all pairwise condition comparisons for the binary measures were nonsignificant. For the conditional duration measures, the only significant adjusted pairwise differences were between V–O phrases and QSWs, for both first-fixation duration and dwell time.

#### 3.2.3. Exploratory Analyses of Pre-Target Regions

To examine whether condition-related differences emerged before the target second component was reached, we analyzed two pre-target regions: the Verb region and the intervening-element region.

In the Verb region, condition did not significantly affect either any-fixation probability, χ^2^(2) = 3.94, *p* = 0.139, or first-fixation duration, χ^2^(2) = 4.80, *p* = 0.091. Tukey-adjusted pairwise comparisons showed no reliable differences among SW, VO, and QSW for either measure. Thus, there was no clear evidence of condition-related processing differences at the first verbal component.

In the intervening-element region, condition effects were significant for both any-fixation probability, χ^2^(2) = 10.31, *p* = 0.006, and first-fixation duration, χ^2^(2) = 8.03, *p* = 0.018. Importantly, SW and VO did not differ reliably for either measure. QSWs showed higher any-fixation probability than both SWs and V–O phrases, and the first-fixation-duration effect was driven primarily by the VO–QSW contrast. These results suggest that QSW-related difficulty may have begun before readers reached the second component, possibly reflecting early contextual uncertainty or parafoveal-preview-related difficulty. Crucially, however, separable words did not differ reliably from V–O phrases in either pre-target region.

#### 3.2.4. Exploratory Sensitivity Analyses

Because QSW sentences were accepted on approximately half of the trials, we conducted exploratory sensitivity analyses to examine whether target-region eye movements were associated with participants’ subsequent acceptability judgments. Among the cleaned QSW eye-tracking trials, 435 (51.4%) were judged acceptable and 412 (48.6%) were judged unacceptable. All 30 participants contributed both accepted and rejected QSW trials; all 30 QSW items had accepted trials, and 29 had rejected trials.

Within the QSW condition, mixed-effects models controlling for standardized log frequency and surprisal showed that subsequently accepted trials had a lower second-fixation probability than subsequently rejected trials, β = −0.604, SE = 0.223, z = −2.71, *p* = 0.007. Accepted trials also had shorter dwell times, β = −0.127, SE = 0.060, t = −2.10, *p* = 0.037. The any-fixation effect was in the same direction and at the conventional significance threshold, β = −0.355, SE = 0.181, z = −1.96, *p* = 0.050, whereas first-fixation duration did not differ clearly by subsequent judgment, β = 0.007, SE = 0.041, t = 0.18, *p* = 0.858. Thus, subsequent acceptability judgments were associated with some target-region processing measures in QSW trials. Because the judgment was made after sentence reading, these analyses should be interpreted as associations between online processing and subsequent judgment, not as evidence that judgment independently caused the eye-movement pattern.

As an additional robustness check, we retained only trials judged acceptable and refitted the additive condition models. In this accepted-only analysis, condition was not significant for any-fixation probability, χ^2^(2) = 0.40, *p* = 0.818, or second-fixation probability, χ^2^(2) = 1.72, *p* = 0.423, but remained significant for dwell time, χ^2^(2) = 6.37, *p* = 0.041, and was marginal for first-fixation duration, χ^2^(2) = 5.69, *p* = 0.058. Tukey-adjusted comparisons showed longer dwell times for accepted QSW trials than for accepted SW trials, SW − QSW = −0.213, *p* = 0.032. These sample-restricted analyses do not replace the primary analyses, but they suggest that the QSW target-region pattern was not entirely attributable to trials that were rejected as unacceptable. Full results are provided in Tables S7 and S8.

## 4. Discussion

The present study compared discontinuous separable words, V–O phrases, and QSWs during sentence reading. Separable words patterned closely with V–O phrases, whereas QSWs showed greater behavioral and target-region processing difficulty descriptively. After frequency and surprisal were added, condition differences in the binary fixation measures were attenuated to non-significance, while the remaining adjusted duration differences were confined to V–O phrases and QSWs.

### 4.1. Processing Similarity and Theoretical Implications for Separable Words

The processing similarity between discontinuous separable words and V–O phrases was evident in both the behavioral and eye-movement results. The two conditions received similarly high acceptance rates and elicited comparable judgment response times. They also did not differ reliably in any-fixation probability, second-fixation probability, first-fixation duration, or dwell time. This similarity was also evident in the adjusted eye-movement analyses: at the mean levels of standardized log frequency and surprisal, none of the pairwise comparisons between separable words and V–O phrases was significant. The present data therefore provide no evidence that discontinuous separable words imposed a stable additional processing cost relative to ordinary V–O phrases.

This similarity is relevant to competing accounts of discontinuous separable words. Lexically oriented analyses derive discontinuous forms from complete lexical units through operations such as copying, movement, or complementary deletion (Chao, 1968; Guo, 2017; Pan & Ye, 2015; Ye & Pan, 2018; Zhao & Zhang, 1996), whereas constructional accounts allow the two components to participate directly in V–O-like syntactic organization (Lü, 1979; Packard, 2000; Zhu, 1982; Cao, 2016; Pang & Zhang, 2022; Yuan, 2018). The present results are compatible with the latter possibility: in the discontinuous contexts examined here, the processing of separable-word components closely resembled that of the corresponding components in ordinary V–O phrases.

This interpretation is consistent with broader evidence that established lexical representations do not preclude access to internal components during processing (Hyönä et al., 2024; Kuperman et al., 2009; Libben, 2007; Lorenz & Zwitserlood, 2014; Taft & Forster, 1975). Research on Chinese compounds has likewise shown that component information contributes to word recognition (Cai et al., 2023; T. Chen et al., 2020; X. Chen et al., 2025). The present findings extend this component-based perspective to discontinuous sentence contexts, showing that separated components can participate in integration even when the expression has a conventionalized lexical representation in its combined form.

The relatively smooth processing of discontinuous separable words may also reflect their distributional and constructional support. Repeated exposure strengthens linguistic representations, whereas predictable material generally incurs lower processing costs (J. L. Bybee, 2013; Staub, 2015). In the present materials, discontinuous separable words were substantially more frequent and less surprising than QSWs, which may have facilitated their integration. Their similarity to V–O phrases may therefore reflect the combined contribution of lexical association, component accessibility, structural integration, and distributional experience.

Taken together, these findings provide online-processing evidence relevant to the morphosyntactic status of Chinese separable words, but they do not resolve the word-versus-phrase debate or imply that separable words are equivalent to freely compositional phrases. In the discontinuous patterns examined here, their components could participate directly in structural integration without a separately measurable cost of restoring the combined lexical form. Chinese separable words may thus be viewed as flexible units whose processing coordinates lexical and constructional knowledge, rather than as categorically word-like or phrase-like on the basis of the present data.

### 4.2. Processing Difficulty of QSWs and Low Distributional Support

Compared with sentences containing separable words or V–O phrases, QSW sentences were accepted substantially less often, elicited markedly longer judgment response times, and showed higher fixation probabilities and longer fixation durations. These descriptive patterns indicate greater processing difficulty for discontinuous QSWs. This pattern should be interpreted cautiously. Because QSW sentences received substantially lower acceptability ratings in the material norming task and were accepted on only about half of the trials in the eye-tracking experiment, the QSW condition should not be treated as a fully acceptable structural counterpart to the separable-word and V–O phrase conditions. Rather, it should be understood as a low-conventionality and low-acceptability comparison condition. Processing difficulty in this condition may therefore reflect not only limited distributional support but also perceived structural markedness, anomaly detection, or uncertainty about whether the separated form is acceptable.

The behavioral and eye-movement findings were consistent with a role for limited distributional support, although they do not show that distributional support was the sole source of QSW difficulty. Within the QSW condition, higher target-structure frequency was associated with shorter judgment response times and a greater probability of an acceptable judgment. Evidence that the frequency association differed across conditions was stronger for judgment response time than for acceptability, because the joint interaction was not significant in the acceptability model. In the binary eye-movement analyses, the omnibus condition effects were attenuated to non-significance after frequency and surprisal were added, and none of the adjusted pairwise condition comparisons was significant. Given the close association between condition and distributional support in the present materials, this attenuation should be interpreted as reflecting shared variance between structural condition and distributional predictors, rather than as evidence that frequency and surprisal fully accounted for the condition differences. Higher frequency was associated with a lower probability of any fixation, whereas higher surprisal was associated with a greater probability of a second fixation.

The exploratory sensitivity analyses also showed that QSW trials subsequently judged unacceptable were associated with greater target-region processing difficulty than trials judged acceptable, particularly in second-fixation probability and dwell time. This finding supports the view that QSW processing was related to participants’ eventual acceptability judgments. However, the accepted-only robustness check also showed that the QSW pattern was not wholly eliminated after rejected trials were excluded, especially for dwell time. Thus, the QSW findings should not be reduced simply to participants’ rejection of unacceptable sentences. Rather, they appear to reflect a combination of low conventionality, reduced acceptability, and limited distributional support.

The conditional duration results were more mixed. After the distributional predictors were added, the overall condition effect remained significant for first-fixation duration but not for dwell time. At the mean predictor levels, V–O phrases showed shorter first-fixation durations and dwell times than QSWs, whereas separable words did not differ reliably from either condition. Thus, the adjusted analyses provide only partial evidence that QSWs retained a processing disadvantage after the distributional predictors were included.

One plausible source of QSW difficulty is the mismatch between a conventionalized combined-form representation and a weakly established discontinuous pattern. Like typical separable words, QSWs are lexicalized disyllabic units. Unlike typical separable words, however, they occur predominantly in their combined forms and provide little established experience of discontinuous realization (Wang, 2011; Zhuang, 2020). Their difficulty may therefore reflect not only limited distributional support for the discontinuous pattern, but also a strong lexical bias toward the combined form. When a QSW is separated, readers may have to accommodate its components within a V–O-like structure despite limited experience with that use. Usage-based and constructional approaches predict that repeated exposure strengthens both lexical representations and particular form–meaning patterns (J. Bybee, 2010; J. Bybee & Beckner, 2010; Goldberg, 2006, 2019). For QSWs, the established combined representation and the weakly licensed discontinuous pattern may therefore create competing processing pressures.

This competition may also help explain why QSWs were not uniformly rejected: approximately half of the experimental trials received acceptable judgments. Some readers may have accommodated the separated components through the available V–O-like pattern, whereas others may have been more strongly constrained by the conventionalized combined form. The present data cannot determine whether these responses reflect different integration strategies, individual differences in constructional experience, or item-specific variation.

Taken together, the QSW contrast helps clarify the smoother processing of typical separable words in two respects. First, typical separable words receive substantially stronger distributional and constructional support for discontinuous realization. Second, their components appear able to enter V–O-like structural integration directly, without an obligatory reconstruction of the combined lexical form. QSWs, by contrast, have weaker support for discontinuous use and may remain more strongly constrained by their conventionalized combined representations. These differences may jointly contribute to the contrasting processing profiles of the two types.

### 4.3. Limitations and Future Directions

Several limitations should be noted. First, structural category, distributional support, and acceptability were closely related in the present materials. This was especially true for the QSW condition, because limited discontinuous use and reduced acceptability are part of what distinguishes QSWs from typical separable words. The additional item-level diagnostics confirmed that condition was strongly associated with both log frequency and surprisal, with limited overlap in the distributional predictor space. The reduction in condition effects after frequency and surprisal were added should therefore be interpreted as evidence of shared variance between condition and distributional support, not as a causal demonstration that distributional support alone accounted for the condition differences. Future studies could address this issue by manipulating discontinuous exposure experimentally, sampling items with greater overlap in frequency and surprisal across conditions, or comparing low-frequency but acceptable separable forms with clearly anomalous pseudo-forms.

Second, the target region was typically a single Chinese character and was frequently skipped. Binary fixation measures were based on all retained eye-movement trials, whereas duration analyses included only trials with a valid corresponding fixation measure. Future studies could use longer target regions or complementary methods such as ERPs to examine component retrieval and integration more directly.

Finally, the study examined native speakers processing relatively simple discontinuous forms containing a single intervening aspect marker or classifier. The findings may not generalize to more complex separations or to second-language learners, whose lexical and constructional experience may differ.

## 5. Conclusions

Discontinuous Chinese separable words patterned closely with ordinary V–O phrases in acceptability judgments and target-region eye movements, providing no reliable evidence of a stable additional processing cost relative to the V–O baseline. QSWs were accepted less often, elicited longer judgment response times, and showed greater descriptive eye-movement difficulty. After frequency and surprisal were added, the omnibus condition effects and all adjusted pairwise comparisons for the binary fixation measures were nonsignificant. For the conditional duration measures, reliable adjusted differences were limited to V–O phrases versus QSWs; separable words did not differ reliably from either comparison condition. These duration results therefore indicate residual difficulty for QSWs relative to the V–O baseline, rather than a clear additional cost for separable words. Within the QSW condition, higher frequency was associated with shorter judgment response times and a greater probability of an acceptable judgment. Across the binary eye-movement analyses, higher surprisal was associated with a greater probability of a second fixation. Because condition and the distributional predictors differed substantially across the materials, these results do not establish that frequency and surprisal uniquely or causally account for the condition differences. Overall, the smoother processing of discontinuous separable words may reflect both their stronger distributional and constructional support and the ability of their components to enter V–O-like structural integration without obligatory reconstruction of the combined lexical form. The findings do not resolve the morphosyntactic status of separable words, but they indicate that lexical representation and discontinuous constructional experience can jointly support online processing.

## Figures and Tables

**Table 1:** Experimental sentences in three conditions.

Condition	First Clause						
**Separable Word**	*Liming*	*zai*	*shan*	*li*	*chi*	*guo*	*ku,*
	Liming	at	mountain	in	eat	EXP	bitter
	Having endured hardships in this mountain,						
**V** **–** **O Phrase**	*Liming*	*zai*	*shan*	*li*	*zhong*	*guo*	*shu,*
	Liming	at	mountain	in	plant	EXP	tree
	Having planted trees in this mountain,						
**QSW**	*Liming*	*zai*	*shan*	*li*	*fu*	*guo*	*pin,*
	Liming	at	mountain	in	support	EXP	poverty
	Having worked on poverty alleviation in this mountain,						
	**Second Clause**						
	*suoyi*	*hen*	*liaojie*		*zheli*	*de*	*qingkuang.*
	so	very	know		here	DE	condition
	Li Ming is very familiar with the local conditions.						

Note. Within each matched set, the target expression varied across conditions, whereas the second clause was held constant. EXP = experiential aspect; DE = nominal modification marker.

**Table 2:** Descriptive statistics for behavioral measures and corpus-based predictors.

Condition	*N*	Acceptance Rate (%)	RT, *M (SD)*, ms	Log RT, *M (SD)*	Raw Frequency, *M (SD)*	Surprisal, *M (SD)*
SW	881	96.8	532 (624)	6.00 (0.65)	794 (949)	3.89 (2.35)
VO	884	98.2	515 (580)	6.00 (0.63)	1539 (1963)	4.46 (1.78)
QSW	889	49.8	1461 (1425)	6.82 (1.02)	8 (21)	10.28 (2.82)

Note. Behavioral statistics include trials with valid judgment response times of at least 100 ms. Frequency and surprisal are summarized at the item level. Raw frequency refers to the untransformed corpus count of attested discontinuous uses of the target V–N combination with any intervening material. Surprisal was computed for the target second component in the sentence-specific V + m context, where m denotes the actual intervening element used in the experimental sentence. RT = judgment response time; SW = separable word; VO = verb–object phrase; QSW = quasi-separable word.

**Table 3:** Fixed effects of the behavioral models.

Panel A. Log-transformed judgment response time				
**Fixed effect**	**Estimate**	***SE***	***t***	***p***
Intercept	6.039	0.102	59.16	<0.001
VO	−0.095	0.125	−0.76	0.451
QSW	0.170	0.153	1.12	0.267
z-log frequency	0.098	0.089	1.11	0.272
z-surprisal	0.157	0.075	2.10	0.039
z-log frequency × VO	−0.106	0.140	−0.76	0.450
z-log frequency × QSW	−0.521	0.159	−3.28	0.001
z-surprisal × VO	−0.275	0.124	−2.21	0.030
z-surprisal × QSW	−0.084	0.111	−0.76	0.450
Panel B. Acceptability judgments				
**Fixed effect**	**Estimate**	***SE***	***z***	***p***
Intercept	5.296	0.996	5.32	<0.001
VO	1.835	1.451	1.27	0.206
QSW	−2.552	1.225	−2.08	0.037
z-log frequency	0.407	0.636	0.64	0.522
z-surprisal	−0.493	0.518	−0.95	0.342
z-log frequency × VO	−1.118	1.107	−1.01	0.313
z-log frequency × QSW	1.631	1.000	1.63	0.103
z-surprisal × VO	1.062	0.977	1.09	0.277
z-surprisal × QSW	0.366	0.703	0.52	0.603

Note. Panel A reports coefficients from the linear mixed-effects model of log-transformed judgment response time. Panel B reports log-odds coefficients from the binomial-logit model of acceptability. SW was the reference condition. The intercept and distributional-predictor coefficients therefore refer to SW, whereas the VO and QSW coefficients represent differences from SW when both standardized predictors equal zero.

**Table 4:** Descriptive statistics for eye-tracking measures in the target region.

Condition	*N*	Any Fixation (%)	Second Fixation (%)	First-Fixation Duration, *M (SD)*, ms	Dwell Time, *M (SD)*, ms
SW	845	33.4	5.1	186 (69)	227 (156)
VO	839	34.1	6.4	186 (85)	229 (143)
QSW	847	52.3	17.2	209 (90)	316 (228)

Note. Duration statistics are reported in milliseconds and include only trials with a positive value for the corresponding measure. The numbers of observations for first-fixation duration were 280, 285, and 436 for SW, VO, and QSW, respectively; the corresponding numbers for dwell time were 282, 286, and 443.

**Table 5:** Pairwise comparisons for binary fixation measures in the target region.

Measure	Contrast	Difference (Log-Odds Scale)	*z*	*p*
Any-fixation probability	SW − VO	−0.131	−0.46	0.890
Any-fixation probability	SW − QSW	0.183	0.53	0.856
Any-fixation probability	VO − QSW	0.314	0.84	0.676
Second-fixation probability	SW − VO	−0.230	−0.64	0.800
Second-fixation probability	SW − QSW	−0.202	−0.52	0.861
Second-fixation probability	VO − QSW	0.028	0.06	0.998

Note. Pairwise comparisons are based on estimated marginal means from the full interaction models at standardized log frequency = 0 and standardized surprisal = 0. Differences are on the log-odds scale; negative values indicate lower fixation odds in the first condition. *p* values are Tukey-adjusted.

**Table 6:** Pairwise comparisons for conditional fixation durations in the target region.

Measure	Contrast	Difference (Log Scale)	*t*	*p*
First-fixation duration	SW − VO	0.097	1.63	0.233
First-fixation duration	SW − QSW	−0.152	−2.19	0.073
First-fixation duration	VO − QSW	−0.250	−3.31	0.003
Dwell time	SW − VO	0.063	0.74	0.740
Dwell time	SW − QSW	−0.200	−2.00	0.116
Dwell time	VO − QSW	−0.263	−2.43	0.043

Note. Duration models include only trials with a positive value for the corresponding measure. Pairwise comparisons are based on estimated marginal means from the full interaction models at standardized log frequency = 0 and standardized surprisal = 0. Differences are on the log-transformed scale; negative values indicate shorter durations in the first condition. *p* values are Tukey-adjusted.

## Data Availability

The anonymized data and analysis scripts supporting the findings of this study are available from the corresponding author upon reasonable request.

## Supplementary Materials

The following supporting information can be downloaded at https://www.mdpi.com/article/10.3390/bs16081285/s1, Supplementary File S1: Supplementary tables, including Table S1: Fixed effects of the additive models for binary eye-movement outcomes; Table S2: Fixed effects of the additive models for conditional fixation-duration outcomes; Table S3: Item-level descriptive statistics for distributional and norming measures by condition; Table S4: Observed ranges of standardized predictors by condition; Table S5: Descriptive statistics for the pre-target-region analyses; Table S6: Condition effects and Tukey-adjusted pairwise comparisons for the pre-target-region analyses; Table S7: Descriptive statistics and model results for accepted versus rejected QSW trials; and Table S8: Accepted-only sensitivity analysis for target-region eye-movement measures. Supplementary File S2: Critical experimental materials and item-level measures.
