# Supplementary material for: Processing Discontinuous Chinese Separable Words: Evidence from Eye Tracking
Source: Behav Sci (Basel). 2026 Jul 28;16(8):1285. doi: 10.3390/bs16081285 (PMC13509747; doi:10.3390/bs16081285)
Supplement: Supplementary file 1 [file behavsci-16-01285-s001.zip › Supplementary_Materials_S1_Tables.pdf]

Supplementary Materials

Processing Discontinuous Chinese Separable Words: Evidence from Eye Tracking

Table S1. Fixed effects of the additive models for binary eye-movement outcomes

Panel A. Any-fixation probability

Fixed effect	Estimate	SE	*z*	*p*	95% CI
Intercept	-0.589	0.204	-2.90	.004	[-0.988, -0.191]
VO	0.093	0.159	0.58	.561	[-0.220, 0.405]
QSW	0.134	0.290	0.46	.644	[-0.435, 0.703]
z-log frequency	-0.297	0.137	-2.17	.030	[-0.567, -0.028]
z-surprisal	0.159	0.104	1.53	.127	[-0.045, 0.364]

Panel B. Second-fixation probability

Fixed effect	Estimate	SE	*z*	*p*	95% CI
Intercept	-2.799	0.223	-12.57	< .001	[-3.236, -2.363]
VO	0.300	0.223	1.34	.179	[-0.137, 0.736]
QSW	0.270	0.351	0.77	.442	[-0.417, 0.957]
z-log frequency	-0.314	0.173	-1.81	.070	[-0.654, 0.025]
z-surprisal	0.346	0.133	2.61	.009	[0.086, 0.606]

Note. Estimates are on the log-odds scale. SW was the reference condition. Log frequency and surprisal were standardized across the 90 critical items. The any-fixation model included random intercepts for participants and items. The second-fixation model included a participant random intercept only because the estimated item-intercept variance was at the boundary of zero. Coefficient-level p values are Wald tests; the main text reports likelihood-ratio tests for the corresponding fixed effects. SW = separable word; VO = verb-object phrase; QSW = quasi-separable word.

Table S2. Fixed effects of the additive models for conditional fixation-duration outcomes

Panel A. Log-transformed first fixation duration

Fixed effect	Estimate	SE	df	*t*	*p*	95% CI
Intercept	5.151	0.029	201.3	176.27	< .001	[5.094, 5.208]
VO	-0.022	0.032	987.3	-0.68	.498	[-0.084, 0.041]
QSW	0.134	0.057	984.8	2.34	.020	[0.022, 0.246]
z-log frequency	0.019	0.027	987.1	0.69	.490	[-0.034, 0.071]
z-surprisal	-0.002	0.020	987.8	-0.09	.928	[-0.041, 0.037]

Panel B. Log-transformed dwell time

Fixed effect	Estimate	SE	df	*t*	*p*	95% CI
Intercept	5.303	0.045	102.9	118.52	< .001	[5.216, 5.391]
VO	0.004	0.047	126.1	0.08	.936	[-0.088, 0.096]
QSW	0.185	0.084	124.3	2.21	.029	[0.021, 0.349]
z-log frequency	-0.002	0.040	120.7	-0.06	.955	[-0.080, 0.075]
z-surprisal	0.055	0.029	115.1	1.88	.063	[-0.002, 0.112]

*Note. Duration models included only trials with a positive value for the corresponding measure. SW was the reference condition. Log frequency and surprisal were standardized across the 90 critical items. The model for first fixation duration included a participant random intercept only because the estimated item-intercept variance was at the boundary of zero. The model for dwell time included random intercepts for participants and items. Approximate degrees of freedom and unadjusted coefficient-level *p* values were obtained using lmerTest. The main text reports likelihood-ratio tests for the corresponding fixed effects and Tukey-adjusted pairwise comparisons from the full interaction models in Table 6. SW = separable word; VO = verb–object phrase; QSW = quasi-separable word.*

Table S3. Item-level descriptive statistics for distributional and norming measures by condition

Variable	Condition	N	Mean	SD	Median	Min	Max
Raw target-structure frequency	SW	30	794.33	949.26	411.00	10.00	4108.00
Raw target-structure frequency	VO	30	1539.20	1962.69	574.50	36.00	7623.00
Raw target-structure frequency	QSW	30	7.70	21.33	0.00	0.00	109.00
Log frequency	SW	30	5.82	1.55	5.97	2.40	8.32
Log frequency	VO	30	6.60	1.28	6.36	3.61	8.94
Log frequency	QSW	30	0.85	1.37	0.00	0.00	4.70
z-log frequency	SW	30	0.48	0.53	0.53	-0.70	1.34
z-log frequency	VO	30	0.75	0.44	0.66	-0.28	1.55
z-log frequency	QSW	30	-1.23	0.47	-1.52	-1.52	0.09
Surprisal	SW	30	3.89	2.35	3.67	0.36	11.51
Surprisal	VO	30	4.46	1.78	4.42	1.66	7.49
Surprisal	QSW	30	10.28	2.82	11.51	1.56	11.51
z-surprisal	SW	30	-0.62	0.63	-0.68	-1.57	1.43
z-surprisal	VO	30	-0.47	0.48	-0.48	-1.22	0.35
z-surprisal	QSW	30	1.09	0.76	1.43	-1.25	1.43
Familiarity rating	SW	30	6.29	0.50	6.33	4.95	7.00
Familiarity rating	VO	30	6.44	0.34	6.45	5.80	6.95
Familiarity rating	QSW	30	6.26	0.40	6.35	5.35	6.80
Acceptability rating	SW	30	8.91	0.41	8.98	7.85	9.55
Acceptability rating	VO	30	8.72	0.33	8.68	8.05	9.30
Acceptability rating	QSW	30	3.98	1.91	3.28	1.70	8.30

Note. Raw frequency refers to the untransformed corpus count of attested discontinuous uses of the target V–N combination with any intervening material. Log frequency was calculated as log(frequency + 1). z values were standardized across the 90 critical items. Familiarity ratings used a 7-point scale; acceptability ratings used a 10-point scale. SW = separable word; VO = verb–object phrase; QSW = quasi-separable word.

Table S4. Observed ranges of standardized predictors by condition

Condition	min z-log frequency	max z-log frequency	0 within range?	min z-surprisal	max z-surprisal	0 within range?
SW	-0.696	1.338	Yes	-1.571	1.425	Yes
VO	-0.279	1.551	Yes	-1.222	0.345	Yes
QSW	-1.520	0.095	Yes	-1.249	1.425	Yes

Note. The within-range columns indicate whether zero, the grand mean of the standardized predictor, fell within the observed marginal range for that condition. SW = separable word; VO = verb–object phrase; QSW = quasi-separable word.

Table S5. Descriptive statistics for the pre-target-region analyses

Region	Condition	Trial N	Any-fixation n (%)	Skip n (%)	FFD N	FFD M (SD), ms	Dwell M (SD), ms
Verb region	SW	845	627 (74.2%)	218 (25.8%)	621	219.2 (76.5)	229.4 (213.2)
Verb region	VO	839	600 (71.5%)	239 (28.5%)	594	216.7 (80.2)	232.1 (210.5)
Verb region	QSW	847	640 (75.6%)	207 (24.4%)	636	226.3 (82.3)	298.0 (280.1)
Intervening-element region	SW	845	483 (57.2%)	362 (42.8%)	457	209.1 (86.4)	154.2 (187.2)
Intervening-element region	VO	839	470 (56.0%)	369 (44.0%)	438	201.0 (84.9)	144.7 (173.7)
Intervening-element region	QSW	847	539 (63.6%)	308 (36.4%)	519	216.1 (87.4)	227.0 (274.7)

Note. The pre-target-region analyses used the same 2,531 cleaned trials as the main target-region analyses. Any-fixation and skip values are reported as counts and percentages. First-fixation duration and dwell time are reported in milliseconds. FFD = first-fixation duration; SW = separable word; VO = verb-object phrase; QSW = quasi-separable word.

Table S6. Condition effects and Tukey-adjusted pairwise comparisons for the pre-target-region analyses

Panel A. Condition effects

Region	Measure	Chi-square	df	p
Verb region	Any-fixation probability	3.94	2	.139
Verb region	First-fixation duration	4.80	2	.091
Intervening-element region	Any-fixation probability	10.31	2	.006
Intervening-element region	First-fixation duration	8.03	2	.018

Panel B. Tukey-adjusted pairwise comparisons

Region	Measure	Contrast	Estimate	SE	Statistic	p	Scale
Verb region	Any-fixation probability	SW - VO	0.153	0.117	z = 1.31	.391	log-odds
Verb region	Any-fixation probability	SW - QSW	-0.080	0.120	z = -0.67	.780	log-odds
Verb region	Any-fixation probability	VO - QSW	-0.234	0.118	z = -1.98	.118	log-odds
Verb region	First-fixation duration	SW - VO	0.017	0.020	az = 0.85	.671	log-ms
Verb region	First-fixation duration	SW - QSW	-0.027	0.020	az = -1.36	.364	log-ms
Verb region	First-fixation duration	VO - QSW	-0.044	0.020	az = -2.20	.071	log-ms
Intervening-element region	Any-fixation probability	SW - VO	0.053	0.110	z = 0.48	.882	log-odds
Intervening-element region	Any-fixation probability	SW - QSW	-0.290	0.111	z = -2.60	.025	log-odds
Intervening-element region	Any-fixation probability	VO - QSW	-0.343	0.112	z = -3.07	.006	log-odds
Intervening-element region	First-fixation duration	SW - VO	0.046	0.028	az = 1.67	.218	log-ms
Intervening-element region	First-fixation duration	SW - QSW	-0.032	0.027	az = -1.19	.458	log-ms
Intervening-element region	First-fixation duration	VO - QSW	-0.078	0.027	az = -2.88	.011	log-ms

Note. Condition effects were tested by likelihood-ratio tests comparing condition-only mixed-effects models with and without condition. Pairwise comparisons were Tukey-adjusted. Estimates for any-fixation probability are on the log-odds scale; estimates for first-fixation duration are on the log-transformed millisecond scale. For duration models, pairwise comparisons were evaluated using asymptotic z tests. Frequency and surprisal were not included because these predictors were defined for the target second component. az = asymptotic z; SW = separable word; VO = verb-object phrase; QSW = quasi-separable word.

Table S7. Descriptive statistics and model results for accepted versus rejected QSW trials

Measure	Group	Trial N	% / M (SD), ms	Duration N	Estimate	SE	z/t	p	95% CI
Any-fixation probability	Rejected	412	58.3%	--	-0.355	0.181	-1.96	.050	[-0.711, 0.000]
Any-fixation probability	Accepted	435	46.7%	--	-0.355	0.181	-1.96	.050	[-0.711, 0.000]
Second-fixation probability	Rejected	412	21.4%	--	-0.604	0.223	-2.71	.007	[-1.041, -0.168]
Second-fixation probability	Accepted	435	13.3%	--	-0.604	0.223	-2.71	.007	[-1.041, -0.168]
First fixation duration	Rejected	412	207.7 (87.1)	237	0.007	0.041	0.18	.858	[-0.072, 0.087]
First fixation duration	Accepted	435	211.2 (92.7)	199	0.007	0.041	0.18	.858	[-0.072, 0.087]
Dwell time	Rejected	412	341.7 (255.7)	240	-0.127	0.060	-2.10	.037	[-0.245, -0.008]
Dwell time	Accepted	435	285.4 (187.1)	203	-0.127	0.060	-2.10	.037	[-0.245, -0.008]

Note. Judgment effect estimates compare accepted trials with rejected trials, with rejected trials as the reference level. Negative estimates therefore indicate lower fixation probability or shorter duration for accepted trials. Binary estimates are on the log-odds scale; duration estimates are on the log-transformed millisecond scale. Models controlled for standardized log frequency and surprisal. For binary outcomes, the descriptive column reports percentages; for duration outcomes, it reports M (SD) in milliseconds.

Table S8. Accepted-only sensitivity analysis for target-region eye-movement measures

Panel A. Descriptive statistics and condition effects

Measure	Condition	Trial N	% / M (SD), ms	Duration N	Chi-square	df	p
Any-fixation probability	SW	819	33.0%	--	0.40	2	.818
Any-fixation probability	VO	825	34.1%	--	0.40	2	.818
Any-fixation probability	QSW	435	46.7%	--	0.40	2	.818
Second-fixation probability	SW	819	5.0%	--	1.72	2	.423
Second-fixation probability	VO	825	6.3%	--	1.72	2	.423
Second-fixation probability	QSW	435	13.3%	--	1.72	2	.423
First fixation duration	SW	819	185.0 (67.1)	268	5.69	2	.058
First fixation duration	VO	825	185.2 (84.7)	280	5.69	2	.058
First fixation duration	QSW	435	211.2 (92.7)	199	5.69	2	.058
Dwell time	SW	819	220.8 (137.7)	270	6.37	2	.041
Dwell time	VO	825	227.4 (141.1)	281	6.37	2	.041
Dwell time	QSW	435	285.4 (187.1)	203	6.37	2	.041

Panel B. Tukey-adjusted pairwise comparisons for accepted-only analyses

Measure	Contrast	Estimate	SE	z/t	p	95% CI	Scale
Any-fixation probability	SW - VO	-0.102	0.168	-0.61	.817	[-0.497, 0.293]	log-odds
Any-fixation probability	SW - QSW	0.035	0.322	0.11	.994	[-0.719, 0.788]	log-odds
Any-fixation probability	VO - QSW	0.137	0.341	0.40	.915	[-0.663, 0.936]	log-odds
Second-fixation probability	SW - VO	-0.259	0.223	-1.16	.477	[-0.781, 0.264]	log-odds
Second-fixation probability	SW - QSW	-0.332	0.393	-0.84	.675	[-1.252, 0.589]	log-odds
Second-fixation probability	VO - QSW	-0.073	0.413	-0.18	.983	[-1.042, 0.896]	log-odds
First fixation duration	SW - VO	0.020	0.032	0.61	.814	[-0.056, 0.096]	log milliseconds
First fixation duration	SW - QSW	-0.141	0.064	-2.21	.070	[-0.290, 0.009]	log milliseconds
First fixation duration	VO - QSW	-0.161	0.068	-2.38	.046	[-0.319, -0.002]	log milliseconds
Dwell time	SW - VO	-0.003	0.043	-0.08	.997	[-0.104, 0.097]	log milliseconds
Dwell time	SW - QSW	-0.213	0.085	-2.52	.032	[-0.412, -0.014]	log milliseconds
Dwell time	VO - QSW	-0.210	0.090	-2.34	.051	[-0.420, 0.001]	log milliseconds

Note. The accepted-only analysis retained only trials that participants judged acceptable. Condition effects were tested by likelihood-ratio tests comparing additive models with and without condition. Pairwise comparisons were Tukey-adjusted and evaluated at standardized log frequency = 0 and standardized surprisal = 0. This analysis was conducted as an exploratory robustness check and does not replace the primary analyses because it conditions on participants' post-reading responses.
